# Micropulse transscleral cyclophotocoagulation (MP-CPC): efficacy endpoints for the treatment of refractory paediatric glaucoma - a retrospective case-series

**DOI:** 10.1186/s12886-024-03358-0

**Published:** 2024-02-28

**Authors:** Nasser Balbaid, Mohammed AlJodai, Ghadeer Fairaq, Maram AlEnzi, Sami AlShahwan

**Affiliations:** 1 King Khaled Eye Specialist Hospital, Riyadh, Saudi Arabia; 2 Prince Mohammed Bin Abdulaziz Hospital , Riyadh, Saudi Arabia; 3 Prince Sultan Medical Military City, Riyadh, Saudi Arabia; 4 Glaucoma department, King Khaled Eye Specialist Hospital, Riyadh, Saudi Arabia

**Keywords:** Micropulse, Transscleral cyclophotocoagulation, Glaucoma, Paediatric glaucoma, Intraocular pressure

## Abstract

**Background:**

This study evaluates the efficacy and safety of micropulse transscleral cyclophotocoagulation (MP-CPC) in paediatric patients.

**Methods:**

This retrospective case-series recruited 44 eyes for glaucoma patients who were younger than 17 years and were treated with MP-CPC and followed for at least one year. Pre- and post-intervention intraocular pressure (IOP) was compared out to at least one year postoperatively. Success rates at 6 months and 1 year postoperatively were assessed. *P* < 0.05 was considered statistically significant.

**Results:**

There were 35 (79.5%) eyes with a history of glaucoma surgery. IOP decreased statistically significantly from a baseline of 32.7 (standard deviation:8.7 mmHg) to 23.2 (8.6) and 21.7 (7.9) mmHg at the 6 months and 1-year follow-up, respectively (*P* < 0.0001, all comparisons). Overall success was noted in 19 (47.5%) eyes at the 6 months follow-up, and 23 (53.5%) eyes at 1 year.

**Conclusions:**

MP-CPC reduces IOP and the burden of medications in paediatric patients with glaucoma. Additionally, its safety profile favours the use of MP-CPC as an adjunctive modality for refractory glaucoma.

## Introduction

Glaucoma is a major cause of irreversible visual deterioration [1, 2]. In children, glaucoma is categorized as primary or secondary. Primary paediatric glaucoma includes primary congenital glaucoma (PCG) and juvenile open-angle glaucoma (JOAG). Secondary glaucoma may be associated with non-acquired disease (or syndrome), non-acquired ocular anomalies, acquired conditions, or glaucoma following cataract surgery [3, 4]. The mainstay of glaucoma management is the maintenance of the intraocular pressure (IOP) within the therapeutic range because it is the only controllable factor. A normal range of IOP is maintained to avoid progression of the glaucoma-related damage to the optic disc and nerve fibre layer. Treatment options include topical and systematic medications, laser procedures, and surgery to achieve the targeted IOP. The latter methods are more invasive, yet are considered more effective in reaching the maintenance IOP target [5].

To treat refractory glaucoma, various ablative techniques are commonly used. Cyclophotocoagulation (CPC) involves ablation of the ciliary body epithelium and thus reduces the production of the aqueous humour thereby reducing IOP. CPC can be transscleral (TS-CPC) or delivered by using an endoscopic method. TS-CPC is conventionally applied as continuous waveform cyclophotocoagulation (CW-CPC). However, it may cause extensive damage to the ciliary epithelium as the laser is applied continuously yielding significant collateral coagulative destruction of the stroma and the ciliary body muscle [6, 7]. Complications of cyclocryotherapy (a precursor of cyclophototherapy) include, hypotony and phthisis bulbi, uveitis, corneal decompensation, severe pain requiring enucleation, choroidal detachment, macular oedema, ptosis, and, possibly, severe visual loss. The incidence of complications is associated to the extent of cyclotherapy at initial treatment (360°, 180°, and 90°) [8, 9]. To mitigate collateral tissue damage, a modified non-continuous laser procedure called micropulse transscleral photocoagulation (MP-CPC) can be used to treat glaucoma. MP-CPC uses a diode laser (IRIDEX IQ810 Laser Systems, Mountain View, CA, USA) to deliver controllable pulsed laser treatment comprised of short active laser ablation interspersed with rest periods. The rest period allows non-pigmented adjacent tissues to stay below their thermal coagulative threshold thus minimizing potential complications [10]. The efficacy and safety of MP-CPC has been evaluated in Saudi Arabia but mainly in adult glaucoma patients [11]. Abdelrahman et al. compared CW-CPC and MP-CPC treatment outcomes at six months postoperatively on a small sample size [12]. El-hefney et al. used micropulse to treat paediatric glaucoma and reported debatable outcomes on safety and efficacy [13]. However other studies of MP-CPC have reported a reduction in the burden of IOP lowering medications and a good safety profile [10, 11, 14]. Most of those studies are limited by small sample sizes and a shorter duration of follow-up. Further studies with a larger sample, a longer duration of follow-up, and using internationally acceptable outcome criteria are preferred.

The current study investigates the efficacy and safety and success rates of MP-CPC in paediatric glaucoma patients.

## Methods

### Sample size and population

This retrospective case-series evaluated patients younger than 17 years with confirmed diagnoses of glaucoma and treated with MP-CPC at King Khaled Eye Specialist Hospital (KKESH). We assume that the outcomes of micro-pulse laser treatment for refractory glaucoma in this paediatric age group are both efficient and safe. Although this is a case-series, to test the two-sided hypothesis and achieve a 95% confidence interval, 80% power of the study, α error of 0.05, and design effect of 0.5, 42 eyes were adequate to address the research question for this study. Finally, we recruited 44 eyes each treated with MP-CPC with 1-year follow-up. All patients were managed at KKESH with MP-CPC from 2015 to 2020. Data were collected from the KKESH database on clinical indices at the preoperative (baseline) visit, at 6 months, 12 months, and at the last postoperative visit.

Specific inclusion and exclusion criteria were used to avoid selection bias. The inclusion criteria implied patients were presenting for follow-up or were following up with the glaucoma clinic with uncontrolled IOP (> 21 mmHg) or worsening disease despite full medical therapy with at least 3 months of follow-up. To be included in the study, the patients should have attended at least 12 months of follow-up after MP-CPC treatment. Patients had to be within 0–16 years old at the time of the surgery. Patients who had undergone intraocular surgery within 2 weeks before MP-CPC were excluded from the study.

IOP was measured with a Tonopen (Reichert Technologies, Buffalo, NY, USA) or a pneumotonometer. Multiple optometrists or ophthalmologists measured the IOP and MP-CPC was performed by multiple surgeons. The standard of success was based on the guidelines from the World Glaucoma Association (WGA) [15], and complete success was defined as an IOP between 6 and 21 mmHg at 6 months, 12 months, and the last follow-up after MP-CPC without any anti-glaucoma medications or need for additional surgery. If the IOP was controlled by medications, it was considered a qualified success. Cumulative overall success was a combination of complete and qualified success. Failure was defined as increased IOP (> 21 mmHg) with antiglaucoma medications or the need for additional surgical intervention. Safety refers to the absence of sight-threatening complications in the intraoperative and postoperative periods.

### Surgical technique

The Cyclo G6 810 nm infrared diode laser with an MP3 probe (IRIDEX IQ810 Laser Systems, Mountain View, CA, USA) was used with a power of 2500 mW and a duty cycle of 31.3%. The “on” time was 0.5 ms and the “off” time was 1.1 ms per cycle. The patients underwent the procedure under general anaesthesia. Topical anaesthetic was placed in the operative eye just prior to placing the diode laser probe perpendicular to the limbus with firm pressure, the probe was then moved in a continuous sweeping, sliding motion over each quadrant, taking 10 s for each sweep, avoiding sites of that had undergone trabeculectomy or tube shunts or other procedures. To avoid the ciliary neurovascular structures, laser treatment was not delivered to the 3 and 9 o’clock positions. The treatment time was based on preoperative IOP as follows:

< 21 mmHg then 160 s treatment time.

< 30 mmHg then 180 s treatment time.

< 40 mmHg then 200 s treatment time.

< 50 mmHg then 220 s treatment time.

< 60 mmHg then 240 s treatment time.

< 60 mmHg then 260 s treatment time.

### Data analysis

A chart review was performed to collect data on patient demographics and clinical glaucoma indices at baseline as well as at 6 months, 1 year and last postoperative visit. Data were entered in Excel 365® (Microsoft Corporation, Redmond, WA, USA). Data were coded and exported to SPSS version 26.0 (IBM Corp., Chicago, Illinois, USA) and all data management, and coding were performed in this software database. Descriptive analysis was performed and categorical variables are reported as frequencies and percentages. Continuous variables are reported as mean (± standard deviation). Inferential analysis performed using Chi^2^ to detect potential associations between success rates and categorical variables. The Mann-Whitney U test was used to investigate potential associations of continuous variables. The Wilcoxon Signed-ranked test was also used to compare means across different follow-up assessments. Mean and median survival estimates were calculated based on a Kaplan-Meier Survival curve. The confidence interval (CI) level was set to 95% and *P* < 0.05 was considered statistically significant.

## Results

Table 1 presents the demographic and clinical indices of the study sample. A total of forty-four eyes of forty patients were included in the current study. The mean age was 10 (4.8), [range, 1–17] years. The study sample was comprised of 21 (52.5%) males and 19 (47.5) females. There were 28 (63.6%), left eyes and 27 (61.4%) phakic eyes. MP-CPC was performed in all 4 quadrants in 21 (47.7%) eyes.

**Table 1:** Baseline demographic and clinical indices of paediatric patients who underwent micropulse transscleral cyclophotocoagulation

Variable	Category	No. (%)
Age	Mean “years” (SD)	10 (4.8)
Gender	Male	21 (52.5)
	Female	19 (47.5)
Eye	OD	16 (36.4)
	OS	28 (63.6)
Lens status	Phakic	27 (61.4)
	Pseudophakic	6 (13.6)
	Aphakic	11 (25)
Quadrant	2	7 (15.9)
	3	16 (36.4)
	4	21 (47.7)
Glaucoma Diagnosis	Congenital	27 (61.4)
	Trauma	1 (2.3)
	JUG	1 (2.3)
	Sturge-Weber	2 (4.5)
	Neovascular	1 (2.3)
	Post SO and RRD	4 (9.1)
	Aphakic	3 (6.8)
	Axenfeld-Rieger	1 (2.3)
	Pseudophakic	2 (4.5)
	Developmental	1 (2.3)
	Congenital & Aniridia	1 (2.3)

OD: right eye OS: left eye JUG: juvenile uveitic glaucoma RRD: retinal rhegmatogenous detachment

There were 24 (54.5%) eyes with congenital glaucoma (Table 1). A history of previous glaucoma surgery was noted in 35 (79.5%) eyes with solely tube shunt placement in 10 (22.7%) of these eyes, and tube shunt with another intervention in 12 (27.3%) eyes (Table 1). Other ocular comorbidities were detected in 19 (43.2%) of these eyes.

There was no association between age groups and the subtype of glaucoma (*P* = 0.393). Glaucoma subtype and gender were not associated (*P* = 0.433).

Table 2 presents the pre- and post-intervention indices. The IOP decreased statistically significantly from 32.7 (8.7) mmHg at baseline to 23.2 (8.6) mmHg at the 6-month and 21.7 (7.9) mmHg at the 1-year follow-up visits (*P* < 0.0001 both comparisons; Table 2). Compared to baseline, there was a statistically significant decrease at the last follow-up visit to 22.3 (12.5) mmHg (*P* = 0.001; Table 2). The difference in mean IOP at 6 months and one-year follow-up was not statistically significant (*P* = 0.589). This comparison was used to assess stability of the procedure.

**Table 2:** Comparison of pre- and post-intervention glaucoma indices of paediatric patients who underwent micropulse transscleral cyclophotocoagulation

Index	Phase	Mean (SD)	p-value
IOP	Preoperative	32.7 (8.7)	Compared as a baseline
	6 months postoperative	23.2 (8.6)	< 0.0001^**^
	One-year postoperative	21.7 (7.9)	< 0.0001^**^
	Last visit postoperative	22.3 (12.5)	0.001^*^
Visual Acuity	Preoperative	0.65 (0.59)	Compared as a baseline
	6 months postoperative	1.5 (0.86)	< 0.0001^**^
	One-year postoperative	1.4 (0.73)	0.002^*^
	Last visit postoperative	1.4 (0.74)	0.004
Number of antiglaucoma medications	Preoperative	2.9(0.77)	Compared as a baseline
	6 months postoperative	1.95 (1.26)	< 0.0001^**^
	One-year postoperative	2.1 (1.1)	< 0.0001^**^
	Last visit postoperative	2.0 (1.1)	0.008^*^

*denotes statistically significant, *P* < 0.05

**denotes highly statistically significant

The mean number of medications decreased statistically significantly from 2.9 at baseline to 1.95 and 2.1 at 6 months and 1-year follow-up (*P* < 0.0001). There was no statistical change in vision between 6 months and 1 year postoperatively (*P* = 0.956). The number of antiglaucoma medications did not differ between the 6-month and first-year follow-up visits (*P* = 0.535).

Table 3 presents the remaining mean IOP values and the number of antiglaucoma medications on postoperative day 1 (20.8, 1.9), week 1 (20.9, 1.9), month 1 (17.6, 1.8), month 3 (14.9, 1.3), month 9 (22.3, 2.5), respectively. Generally, the values for both variables were statistical significant at all visits compared to the baseline values (Table 3).

**Table 3:** Comparison between pre- and post-intervention IOP and number of medications on designated visits

Follow-up Assessment Visit	IOP Mean (SD)	p-value	No. of Medications Mean (SD)	p-value
Preoperative	32.7 (8.7)	Compared as a baseline	2.9 (0.8)	Compared as a baseline
Day 1 Postoperative	20.8 (9.1)	< 0.0001^**^	1.9 (1.3)	0.002^*^
Week 1 Postoperative	20.9 (8.3)	< 0.0001^**^	1.9 (1.2)	< 0.0001^**^
Month 1 Postoperative	17.6 (10.9)	< 0.0001^**^	1.8 (1.2)	< 0.0001^**^
Month 3 Postoperative	14.9 (13.1)	< 0.0001^**^	1.3 (1.3)	< 0.0001^**^
Month 6 Postoperative	23.2 (8.6)	< 0.0001^**^	1.9 (1.3)	< 0.0001^**^
Month 9 Postoperative	22.3 (6.2)	0.023^*^	2.5 (0.9)	0.005^*^
Month 12 Postoperative	21.7 (7.9)	< 0.0001^**^	2.1 (1.1)	< 0.0001^**^
Last Follow-up visit	19.3 (12.5)	0.002^*^	2 (1.1)	0.008

*denotes statistically significant, *P* < 0.05

**denotes highly statistically significant

**Fig. 1:**
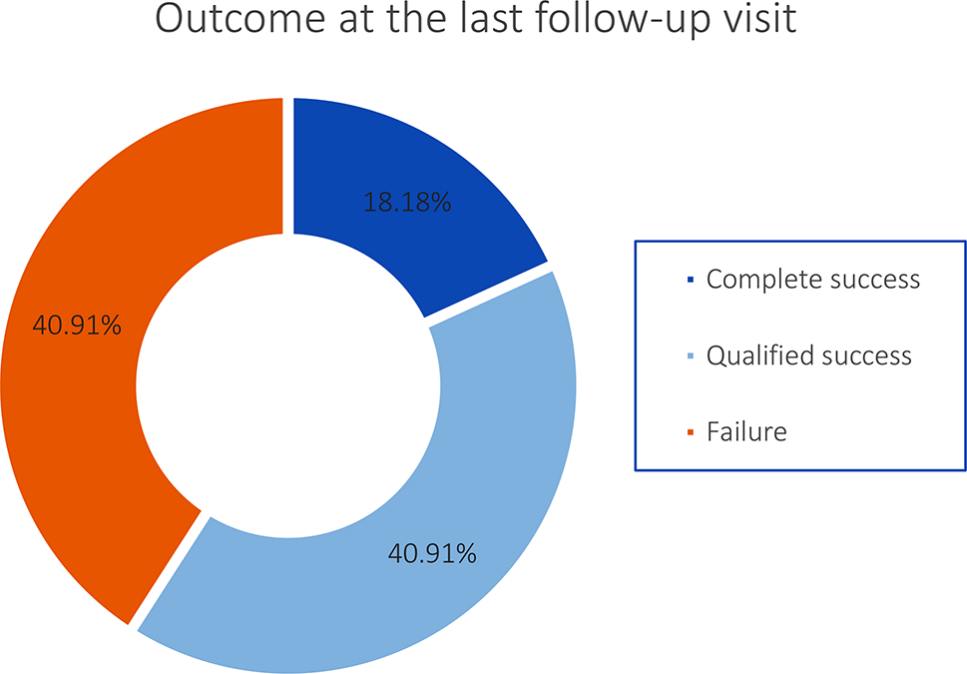
Success rates at the last follow-up visit assessment for paediatric patients who underwent micropulse transscleral cyclophotocoagulation

The overall success rate (complete plus qualified success) was 47.5% (19 eyes) at 6 months, 53.5% (23 eyes) at one-year, and 59.1% (13 eyes) at the last follow-up visit (some patients were lost to follow up) (Table 4; Fig. 1).

**Table 4:** Success rates at different postoperative follow-up assessments among paediatric patients who underwent micropulse transscleral cyclophotocoagulation

Success Rate	M 6 No. (%)	M 12 No. (%)	L.V No. (%)
Complete Success	2 (5)	1 (2.3)	4 (18.2)
Qualified Success	17 (42.5)	22 (51.2)	9 (40.9)
Overall Success	19 (47.5)	23 (53.5)	13 (59.1)
Failure	21 (52.5)	20 (46.5)	9 (40.9)

M6 denotes 6 months visit, M12 denotes 12 months visit, L.V denotes last visit (past 12 months)

There was a statistically significant positive association between the success rate at 1 year and increasing age at surgery (*P* = 0.016). There were no significant associations between success rate and quadrant or lens status (*P* = 0.935 and *P* = 0.488, respectively). Success rate was not associated to: laser power levels (*P* = 0.493), duration (*P* = 0.674), glaucoma subtype (*P* = 0.789), previous glaucoma surgery (*p* = 0.889) and, number of previous glaucoma surgeries (*P* = 0.818).

**Fig. 2:**
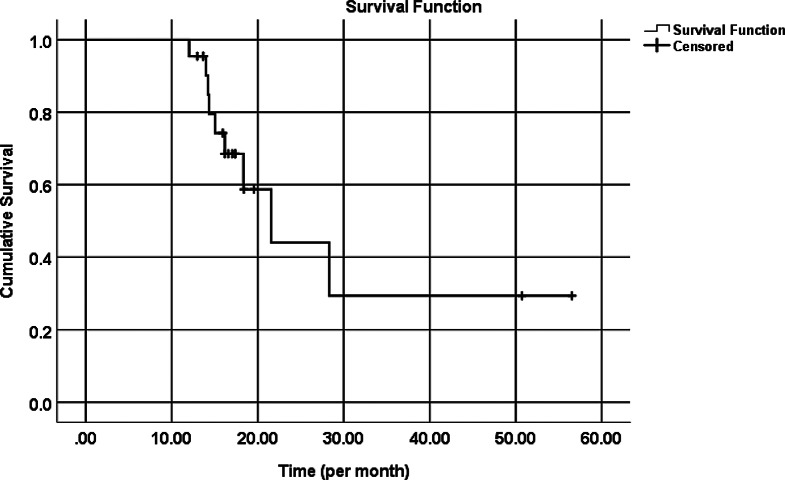
Estimated survivorship plot

Over the course of follow-up, the majority of cases [38 (84.4%) eyes], did not have a major sight-threatening complication (Table 5). Findings from the Kaplan-Meier Survival Curve indicate that mean (SE) survival time was 30.2 (5.7) months [95% CI:19.107–41.378 months], and median (SE) survival time was 21.6 (3.5) [95% CI:14.659–28.481 months] (Fig. 2).

**Table 5:** Procedure-related complications among paediatric patients who underwent micropulse transscleral cyclophotocoagulation

Complication	No. (%)
No complications	38 (86.4)
VA decline	3 (6.8)
Hypotony	2 (4.5)
Neurotrophic ulcer	1 (2.3)
Total	44 (100)

VA denotes visual acuity

## Discussion

This retrospective study allowed an assessment of the role of MP-CPC in the management of refractory glaucoma in paediatric patients. Cyclophotocoagulation in the paediatric age group has been reported using cyclocryotherapy, transscleral cyclophotocoagulation, and endoscopic cyclophotocoagulation. Lee et al. [16], reported varied outcomes of MP-CPC in children. Their [16] study compared MP-CPC in adults and paediatric patients and found that the results were less encouraging in the paediatric group compared to the adult group. In Lee et al’s [16] study, 7 of 9 children who underwent MP-CPC required another IOP-lowering procedure within 1 year postoperatively. They [16] concluded that the low success rate in the paediatric age group (22%) could be due to the higher regenerative ability of the ciliary body in children or due to the smaller area of treatment (2 quadrants). Abdelrahman et al. [12] and El-hefney et al. [13] reported more promising results with success rates of 71% and 61% respectively. Both groups attributed the success rates to the greater extent of treatment (4 quadrants).

The introduction of MP-CPC may increase the role of cyclodestructive procedures. Cyclodestructive procedures were originally used if the medical therapy was adequate for controlling IOP and the patient and other factors precluded the patient from invasive surgery [17]. Although satisfactory outcomes and safety have been reported with MP-CPC for refractory glaucoma in adults [10, 18], there is a paucity of studies on its role in the paediatric population. An advantage of MP-CPC is its repeatable and titratable nature. Some surgeons prefer to increase the duration of the laser treatment to increase efficacy, while others prefer to increase laser power to achieve a more acceptable IOP, especially in eyes with extremely high IOP.

The mode of action of diode micropulse laser is the photocoagulation of the pigmented ciliary epithelium that permits cooling during rest periods between pulses, which minimizes collateral damage to the adjacent tissue. In contrast to continuous mode TS-CPC, the micropulse laser does not clinically manifest tissue disruption (the pop sound heard with TS-CPC) during laser delivery [9].

In our study, the overall success rate was 53.5% at 1 year and 59.1% at the last follow-up visit. We believe these are promising outcomes with a less invasive procedure. In our study, on the first postoperative day, some patients had low IOP and others experienced spiking IOP. These differences in patient presentation were likely related to the different responses of the ciliary body and the inflammatory process of the individual patient’s eye [10]. We found that following the initial reduction or elevation during the first week, the IOP begins to decrease to normal levels or returns to the pre-op IOP level throughout the follow-up period.

The main concern with cyclodestructive procedures is the potential risk of vision-threatening complications, such as inflammation, hypotony, choroidal detachment, macular oedema, sympathetic ophthalmia, and phthisis bulbi. Hence, surgeons are hesitant to routinely use these procedures and tend to reserve them for eyes with very poor vision. Williams et al. [19], reported a relatively high rate of complications in their study on MP-CPC in refractory glaucoma, when they used a *“stop-and-go”* pattern for laser application, they reported hypotony in 8.8% of eyes, corneal oedema in 2%, prolonged anterior chamber reaction for greater than 3 months in 26%, and phthisis in 2% of eyes. In the current study, we applied the micropulse laser for a duration of 40 to 120 s based on the preoperative IOP. The laser was delivered in a continuous slow sliding motion. In the current study, we encountered only two cases of hypotony and a safety profile of 87% which is encouraging. These outcomes concur with Abdelrahman et al’s report [12] of one case of hypotony in 17 eyes that underwent MP-CPC that resolved spontaneously without requiring medical therapy for IOP control.

The lower risk of complications with MP-CPC reported in the current study is consistent with published literature. For example, Lee et al. [16], did not encounter any complications in paediatric eyes treated with MP-CPC, apart from some early mild inflammation.

Kaplan-Meier survival analysis indicated failure in 9 cases that began between 1 year and 1.5 years postoperatively. This would broaden our horizon to the opportunity for reoperating trials.

Our study has some limitations including the inconsistent follow-up over the duration of this study that spanned the COVID-19 pandemic which made some patients reluctant to visit the hospital. Hence data were missing data in some cases with no variables documented except for medication refill and complications reported through telephone interviews. This also limited the scope of the study as some of the variables we had originally included did not get well documented, and therefore were excluded. However, given the scant publications on MP-CPC in paediatric cases, we believe this study provides important insights that can aid clinicians and surgeons in managing glaucoma.

In conclusion, the outcomes of this study indicate that MP-CPC may reduce IOP and decrease the burden of medications on paediatric glaucoma patients. The outcomes of MP-CPC are affected by the type of glaucoma, lens status, number of quadrants, different laser settings, or the number of previous glaucoma surgeries. The safety profile favours the use of MP-CPC as an adjunct for managing refractory glaucoma.

## Data Availability

The datasets used and/or analysed during the current study are available from the corresponding author upon reasonable request.
